# Geometric Morphometric Assessment of Toe Shape in Forest and Urban Lizards Following Hurricane Disturbances

**DOI:** 10.1093/iob/obad025

**Published:** 2023-07-12

**Authors:** R Michaud, T J Hagey, L F De León, L J Revell, K J Avilés-Rodríguez

**Affiliations:** Department of Biology, University of Massachusetts Boston, 100 Morrissey Blvd, Boston, MA 02125, USA; Department of Science and Mathematics, Mississippi University for Women, 1100 College Street, Columbus, MS 39701, USA; Department of Biology, University of Massachusetts Boston, 100 Morrissey Blvd, Boston, MA 02125, USA; Department of Biology, University of Massachusetts Boston, 100 Morrissey Blvd, Boston, MA 02125, USA; Department of Biology, University of Massachusetts Boston, 100 Morrissey Blvd, Boston, MA 02125, USA; Louis Calder Biological Field Station-Fordham University 31 Whippoorwill Rd, Armonk, NY, USA

## Abstract

Evidence suggests that hurricanes can influence the evolution of organisms, with phenotypic traits involved in adhesion, such as the toepads of arboreal lizards, being particularly susceptible to natural selection imposed by hurricanes. To investigate this idea, we quantified trait variation before and after Hurricanes Irma and Maria (2017) in forest and urban populations of the Puerto Rican lizard *Anolis cristatellus*. We found that the hurricanes affected toe morphology differently between forest and urban sites. In particular, toepads of the forefeet were longer and narrower in forest, but wider in urban populations, compared to pre-hurricane measures. Toepads of the hind feet were larger in area following the hurricanes. Fore and rear toes increased in length following the hurricane. There were no changes in the number of lamellae scales or lamellae spacing, but lamellae 6–11 of the forefeet shifted proximally following the hurricane. We also measured clinging performance and toe shape. We found that toepad area and toe lengths were stronger predictors of adhesive forces than toepad shape. Our results highlight an interaction between urbanization and hurricanes, demonstrating the importance to consider how urban species will respond to extreme weather events. Additionally, our different results for fore and rear feet highlight the importance of evaluating both of these traits when measuring the morphological response to hurricanes in arboreal lizards.

## Spanish Abstract

La evidencia sugiere que los huracanes pueden influir en la evolución de los organismos, rasgos fenotípicos como las almohadillas distales de los lagartos arbóreos, son particularmente susceptibles a la selección natural impuesta por los huracanes. Para investigar esta idea, cuantificamos la variación de las almohadillas distales antes y después de los huracanes Irma y María (2017) en poblaciones de bosques y urbanas de las lagartijas puertorriqueña *Anolis cristatellus*. Encontramos que los cambios morfológicos luego de los huracanes variaron entre las poblaciones de bosque y urbanas. Para las poblaciones de bosque, las almohadillas de las patas delanteras eran más largas y estrechas luego de las tormentas. Por el contrario, las almohadillas delanteras de las poblaciones urbanas fueron más anchas luego de los huracanes. Las almohadillas de los pies traseros tenían un área más grande en todas las poblaciones luego de los huracanes. Los dedos delanteros y traseros aumentaron de longitud después del huracán. No hubo cambios en el número de escamas en las almohadillas distales ni en el ancho de estas escamas, pero las escamas 6–11 de las patas delanteras se desplazaron proximalmente después del huracán. También medimos las fuerzas adhesivas producida por las almohadillas distales para contrastar con la morfología de los dedos. Encontramos que el área de las almohadillas distales y la longitud de los dedos fueron los predictores más fuertes de las fuerzas adhesivas. Nuestros resultados destacan una interacción entre la urbanización y los huracanes, lo que demuestra la importancia de considerar cómo responderán las especies urbanas a los eventos climáticos extremos. Además, nuestros diferentes resultados para las patas delanteras y traseras resaltan la importancia de evaluar ambos rasgos al medir la respuesta morfológica a los huracanes en lagartijas arbóreos.

## Introduction

The global climate change crisis has spurred interest in the ecology and evolution of species facing extreme weather events (Urban et al. 2016; Kingsolver and Buckley 2017; Radchuk et al. 2019). Indeed, climate models predict increased intensity and severity of extreme weather events, including hurricanes (Bender et al. 2010; Bhatia et al. 2013). Hurricanes are powerful storms that can modify ecosystems and ecological communities (Waide 1991; Lugo 2008; Zimmerman et al. 2014). One such disturbance is canopy shearing, which can result in short- and long-term community changes (Willig, Presley, and Bloch 2011; Zimmerman et al. 2021). For example, an abundance of pioneer plant species resulted in increased population sizes of walking sticks (*Lamponius sp.*), but also increased mortality due to the higher temperatures associated with a reduction of canopy cover (Willig et al. 2011). As such, some studies have begun to apply ecological and molecular tools to understand if species can endure or adapt to extreme weather events (Campbell-Staton et al. 2017; Siepielski et al. 2017; Donihue et al. 2018; Dufour et al. 2019; Riddell et al. 2021).

In addition to impending climate change, many species face challenges owing to anthropogenic habitat loss and modification and the human-facilitated spread of non-native species and diseases (Alberti et al. 2020; Miles et al. 2021). Interestingly, some species can thrive in heavily urbanized areas, exploiting beneficial conditions, such as the lower abundance of competitors or predators (Pieniążek, Sokół, and Kozakiewicz 2017), or the presence of new habitats, such as artificial night lighting (Schoeman 2016; Baxter-Gilbert et al. 2021; Nordberg and Schwarzkopf 2022). An urban environment can provide novel resources, such as supplemental feeding opportunities (Stofberg et al. 2019). Moreover, some urban exploiters have not only acclimated but adapted to urbanization, resulting in replicated patterns of phenotypic change across multiple cities (Diamond et al. 2018; Cosentino and Gibbs 2022; Santangelo et al. 2022). For example, white clover (*Trifolium repens* L., Fabaceae) populations evaluated across 160 cities tended to produce less hydrogen cyanide, a chemical related to water stress and herbivory resistance, compared to non-urban counterpart populations (Santangelo et al. 2022). Similarly, in some species of Darwin's finches, human food subsidy exploitation reduces the correlation between seed toughness and beak morphology (De León et al. 2011, 2019). Due to the scarcity of studies of highly urbanized populations responding to climate disturbances, it remains unclear how the effects of urbanization and extreme weather events such as hurricanes may interact to drive evolutionary change: climate disturbances may affect urban and forest populations differently.

In 2017, Hurricanes Irma and Maria, both Category5 storms, impacted several island countries in the Caribbean (Pasch, Penny, and Berg 2018). Subsequently, multiple studies used tropical anoles (lizards in the genus *Anolis*) as a model to evaluate evolutionary responses to hurricanes (Donihue et al. 2018, 2020; Dufour et al. 2019; Rabe et al. 2020; Avilés-Rodríguez et al. 2021; Simon et al. 2023). In particular, focusing on the Turks and Caicos and Florida (USA), Donihue et al. (2018, 2020) and Rabe et al. (2020) both found significant phenotypic shifts in anole limb and toepad morphology following the hurricanes. Laboratory experiments showed that anoles with longer forelimbs, larger toepads, and shorter hindlimbs had a lower probability of dislodgement from their perches (Donihue et al. 2018). Lizards with longer forelimbs had a stronger gripping ability; however, lizards with longer hindlimbs were more likely to be dislodged by wind exposure (Debaere et al. 2021).

Anole toepads are also likely targets of hurricane-driven selection due to their tight association with structural habitat use (Irschick et al. 1996; Miller and Stroud 2021). Anole toepads are composed of modified ventral lamellae scales that are covered in microscopic hair-like structures called setae. Adhesion is achieved by Van der Waals bonds between setae and any surface to which an anole clings (Williams and Peterson 1982; Autumn and Peattie 2002; Garner et al. 2019). Prior research has shown that anoles with larger toepads and more lamellae produce greater clinging forces (e.g., Irschick et al. 1996). Larger toepads have also been associated with more arboreal habitat use (Elstrott and Irschick 2004), and the evolution of toepads is thought to have contributed to species diversification (Burress and Muñoz 2022). The evaluation of morphological responses to Hurricane Maria in anoles have shown that hurricanes can sometimes drive rapid phenotypic change, but the magnitude and direction of this change can vary substantially depending on the species, island, and habitat type. For instance, research by Avilés-Rodríguez et al. (2021) showed that following the 2017 hurricanes, populations of Puerto Rician *Anolis cristatellus* decreased in average body size and relative toepad area in forest areas—while urban populations decreased in body size, but largely maintained the same toepad morphology relative to their body size. Moreover, Dufour et al. (2019) found no morphological shifts associated with Hurricane Maria in two anole species (*A. cristatellus, A. oculatus*) on the island of Dominica. Similarly, populations of *Anolis gundlachi* did not show changes in body size or limb lengths in response to the 2017 hurricanes in Puerto Rico (Acevedo et al. 2022).

Anole toepads have also been demonstrated to be under selection in urban habitats (Winchell et al. 2016, Winchell 2018). In particular, urban anoles tend to have larger toepads and longer limbs: Traits that have been hypothesized to improve clinging performance when perching on smooth surfaces typical of urban habitats. Moreover, larger toepad areas can compensate for the loss of functional utility of the claw on anthropogenic substrates, such as painted concrete (Naylor and Higham 2019; Falvey et al. 2020), and contribute to faster sprint speed during locomotion on inclined smooth substrates (Winchell et al. 2016, 2018). In addition, Howell, Winchell, and Hagey (2022) showed that *Anolis* toepads tend to change in shape and overall size in lizards that have colonized the urban realm.

We used geometric morphometrics to evaluate how urbanization and hurricanes may have affected toepad size and shape in lizards of the Puerto Rican species, *A. cristatellus*. Specifically, we assessed variation in toe and toepad associated with hurricanes, beyond changes in area and total length. Furthermore, we evaluated how this toe and toepad variation may be linked with clinging performance. This study will help illuminate and predict morphological responses to hurricanes and urbanization, both increasingly important phenomena on our changing planet.

## Methods

### Ecological context of Caribbean 2017 hurricanes

In September of 2017, the island of Puerto Rico endured two Category 5 hurricanes. First, Hurricane Irma passed north of the island without making landfall, but nonetheless causing major wind and rainfall in Puerto Rico. Later that same month, Hurricane Maria made landfall near the Municipality of Yabucoa and exited the northwestern side of Puerto Rico near the municipality of Arecibo days later. These two hurricanes caused extensive structural habitat changes, including beach erosion, forest canopy shearing, tree mortality, landslides, and changes to the makeup of multiple species communities (Hu and Smith 2018; Barreto-Orta et al. 2019; Meléndez-Vazquez et al. 2019; Bessette-Kirton et al. 2020; Pérez Valentín and Müller 2020).

### Field collection

We collected *A. cristatellus* lizards at four sites in the municipality of San Juan, Puerto Rico, once before and twice after the September hurricanes (Table 1). We collected at two forest sites: Bosque del Nuevo Milenio (BNM) and Bosque San Patricio (BSP), and at two urban sites: The University of Puerto Rico Rio Piedras (UPR) and the urban park Parque Adolfo Dones (PAD). Our sampling took place at yearly intervals during the dry, non-reproductive season (December–February). Access to all sites was not possible at all sampling time points. All sites were sampled 7 months before the hurricanes in February 2017 as part of different research. A total of 16 months later, in January 2019, we sampled three sites (BSP, UPR, and PAD). At this time, the forest BNM was closed to all visitors and staff due to hurricane damage. Lastly, 27 months after the hurricane, in December 2019, we sampled three sites (BNM, UPR, and PAD). At this time, the forest BSP was closed to all visitors and staff.

**Table 1:** Sample sizes of lizards per site per date.

Hurricane context	Habitat	Site	Sample size
7 months before	Forest	BNM	44
7 months before	Forest	BSP	39
7 months before	Urban	PAD	42
7 months before	Urban	UPR	32
16 months after	Forest	BNM	0*
16 months after	Forest	BSP	25
16 months after	Urban	PAD	26
16 months after	Urban	UPR	25
27 months after	Forest	BNM	52
27 months after	Forest	BSP	0*
27 months after	Urban	PAD	27
27 months after	Urban	UPR	35
Total			347

*Note*: *Forest sites were not accessible at all times due to logistical constraints and staffing shortages of forest areas after the hurricane.

Male adult *A. cristatellus* lizards were collected during the day using collapsible poles with slip knots. We collected an average of 30 lizards per site per time point for a total of 347 individuals (Table 1). Captured lizards were transported to a nearby field laboratory where morphology and performance measurements were collected. We measured lizard body length as the distance between the tip of the snout and the vent (SVL) using a plastic ruler. We measured body mass using a digital scale (±0.01g). We collected data on clinging performance by suspending a clipboard attached to a force transducer at a near vertical angle (∼5° from vertical due to a small displacement associated with suspending the clipboard). A clean acetate sheet was attached to the clipboard. Lizards were held at the pelvis, placed on the acetate sheet such that all four feet were contacting the sheet and then gently pulled downward, parallel to the clipboard, until their front limbs were fully extended. Lizards were then pulled 2–5 cm farther along the acetate surface, to ensure extension of the rear feet. Thus, we quantified the contact of all four feet to evaluate their maximum frictional forces generated via their adhesive toepads. Our force transducer (Extech 475040) displayed the maximum force in Newtons (accuracy 0.4%). If a limb became dislodged during the trial, we ended and repeated the trial. We retested each lizard until they performed three acceptable trials or after 10 consecutive failed attempts in which the lizard did not engage their toepads. Lastly, we imaged each lizard's feet. After performance trials, but before imaging, lizards were anesthetized using a small aerial dosage of isoflurane to minimize image distortion due to lizard movement. We used a flatbed digital scanner (Epson V300) to take high resolution scans of the lizards’ fore and hind feet (at 2100 dpi). All images included a ruler for scale. Lizards were allowed to recover from anesthesia overnight and then returned to their location of capture the next morning.

### Image processing

**Fig. 1:**
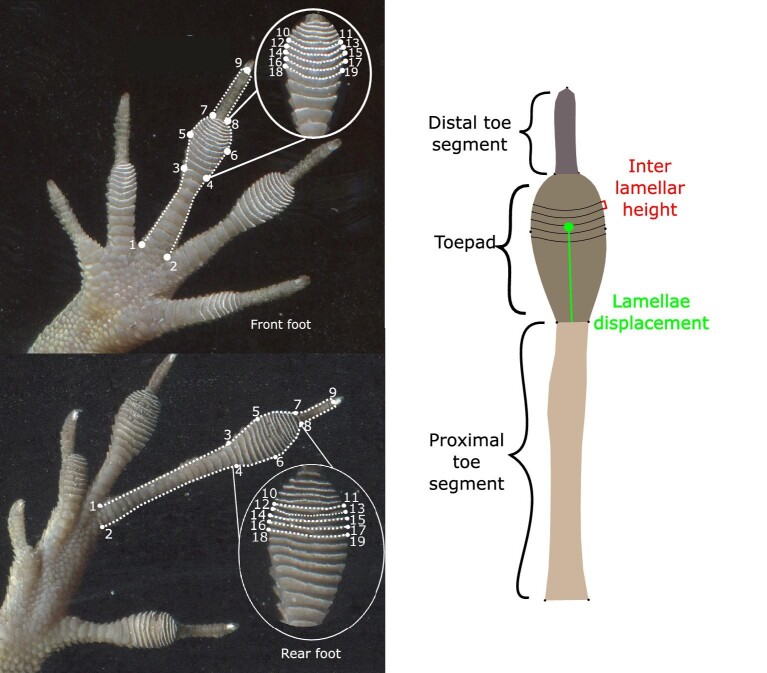
Placement of landmarks and curves on front and rear feet of *Anolis cristatellus* (left image). Landmarks one through nine-capture toe shape. Landmarks 10–19 capture lamellae morphology. The illustration of a rear toe (right image) highlights the proximal toe segment, toepad, and distal toe segment in increasingly darker tones of brown. Interlamellar height in red is the average Euclidean distance between landmarks 10 & 12, 11 & 13, 12 & 14, 13 & 15, 14 & 16, 15 & 17, 16 & 18, and 17 & 19. Lamellae displacement in green denotes Euclidean distance between the Euclidean center of landmarks 10–19 (i.e., the center of mass of lamellae 10 through 19) and the midpoint between lamellae 3 & 4.

Toepad shape data for geometric morphometric analyses were collected from our flatbed scans using the landmarking programs tpsUtil and tpsDig2 (Rohlf 2006, 2015). We gathered data from both the rear and fore feet for each individual as two separate shape datasets (Fig. 1). Focal left or right feet were chosen based on their positioning on the scanner (e.g., straight and flat toes against the scan surface), and thus our dataset contains a mixture of right and left feet. For consistency, we mirrored our right feet images, so they appeared as left feet prior to landmarking. We focused on the largest toe of the forefoot (the third toe) and hind foot (the fourth toe) of each lizard (Fig. 1) and followed a similar landmark scheme as in Howell et al. (2022). We placed landmarks 1 and 2 at the left and right base of the toe where it meets the palm (Fig. 1). Landmarks 3 and 4 were placed on the left and right sides of the proximal base of the toepad where the toe begins to widen. Landmarks 5 and 6 were placed on the left and right widest points of the toepad. Landmarks 7 and 8 were placed on the left and right distal end of the toepad, and landmark 9 was placed on the ventral proximal base of the claw. Landmarks 10–19 were placed on the left and right distal edges of lamellae 6–11 (counting distal to proximal). We chose these specific lamellae scales, as they are large enough to be easily landmarked and typically occur near the widest region of the toepad. In addition, distal lamellae may be developmentally homologous (Griffing et al. 2022) across species. We then connected our landmarks using curves to outline the silhouette of the toe, connecting landmarks 1–3, 3–5, 5–7, 7–9, 9–8, 8–6, 6–4, and 4–2. We also placed a curve along the distal free end of lamellae 6–11, connecting landmarks 10 and 11, 12–13, 14–15, 6–17, and 18–19. Each curve initially included 10 equally spaced semilandmarks, although the first and last semilandmarks were later removed because they overlapped with existing landmarks, for eight semilandmarks per curve. Each image included a ruler, which was used to calculate a scale factor.

### Statistical analyses

We used R (version 4.1.2) and Rstudio (version 1.41) (R Core team 2019; Posit team 2023) to perform all of our statistical analyses. Using the R package *geomorph 4.0.1* (Baken et al. 2021), we conducted a Procrustes analysis to align our landmark data and remove size, treating our front and rear toepad shape data as two independent datasets. Semilandmark location was optimized by minimizing their bending energy. We then verified our landmark placement by identifying outlying individuals using the R function *plotOutliers* (Baken et al. 2021). To identify specific misplaced landmarks, we also calculated the Euclidean distance of each landmark from the mean location of that landmark across specimens. An individual specimen's landmark was flagged as an outlier, if its distance from its mean was greater than the upper quartile. Images containing outlier landmarks were reviewed to verify correct landmark placement. In the cases of misplaced landmarks, images were re-landmarked. In other cases, toes with multiple outlying landmarks were bent and thus, either the opposite foot was landmarked (e.g., left instead of right) or if both feet were unacceptable for landmarking, individuals were removed from the dataset. After the removal or re-landmarking of individuals, we reran our Procrustes and outlier analyses until no further misplaced landmarks were identified. Our final sample size was 326 individuals with fore toe shape data and 302 individuals with hind toe data.

### Toe shape in response to hurricanes

We first tested whether front or rear toepad shape varied significantly in the months before and after the hurricanes **for each of our four sites independently** using a type-2 Procrustes analysis of variance (ANOVA) (function *procD.lm*, (Baken et al. 2021)) on our aligned fore and hind datasets. Our model treated front or rear toepad shape as a dependent variable, with our independent “Hurricane” variable treated as a numerical value, capturing the number of months after the landfall of Hurricane Maria (−7 months before, +16 months after, and +27 months after). We tested sites independently since we could not access every site at every time point after the hurricane. We visualized the results of the Procrustes ANOVA by extracting the regression scores of the model and plotting a histogram of these values. The regression scores represent the shape vector for each individual, denoting how shape diverged from the shape predicted by the linear model stated above. We then conducted principal component analysis (PCA) to visualize the general patterns in our front and rear datasets (using the *gm.prcomp* function in the geomorph package) and their relationships to our hurricane and habitat (urban or forest) variables. For both our PCA and Procrustes ANOVA analyses, we generated projected toe shapes to visualize the relevant relationships. For our ANOVAs, we generated the maximum and minimum shape along the regression line of each linear model. For our PCA, we generated maximum and minimum shape projections for each PC axis. To confirm any significant correlations with toe shape, we evaluated post-hoc linear models using univariate morphological measurements.

### Univariate morphology

We generated linear measurements using our landmark data to test specific hypotheses of how toe shape may have changed in response to hurricanes, motivated by our Procrustes ANOVA results. Using our raw scaled landmark data (i.e., prior to performing a Procrustes alignment of our specimens), we calculated linear distances (in mm) between landmarks to obtain the following measurements (see Fig. 1): toe length (the sum of the Euclidean distances between the midpoint of landmarks 1 & 2, 3 & 4, 5 & 6, 7 & 8, and landmark 9), proximal toe segment length (toe length up to the start of the toepad, between the midpoints of landmarks 1 & 2 and landmarks 3 & 4), toepad length (Euclidean distance between the midpoints of landmarks 3 & 4 and 7 & 8), toepad width (Euclidean distance between landmarks 5 and 6), distal toe segment length (Euclidean distance between the midpoint of landmark 7 & 8 and 9), and average inter-lamellar height (average Euclidean distances between landmarks 10 & 12, 11 & 13, 12 & 14, 13 & 15, 14 & 16, 15 & 17, 16 & 18, 17 & 19; Fig. 1).

We calculated toepad area by measuring the area enclosed by landmarks 3 through 8 and the semi-landmarks connecting landmarks 3–5, and 5–7, 8–6, and 6–4 (Fig. 1). We also calculated the aspect ratio of each specimen's toepad as the toepad width divided by the toepad length, and lamellae displacement as the Euclidean distance between the center of landmarks 10 through 19 and the midpoint between lamellae 3 & 4 divided by the total toepad length. These measurements provide a relative estimate of how distal lamellae 6–11 are on the toepad, with higher values closer to one representing more distally located lamellae. Previous studies have documented shifts in lizard body size (SVL) associated with hurricanes (Donihue et al. 2018; Dufour et al. 2019; Avilés-Rodríguez et al. 2021). To control for specimen body size, we first evaluated whether the hurricane affected the body size of our populations. In the case of finding a shift in body size associated with the hurricanes, we regressed the natural logarithm of each variable (except for ratio variables like lamellae displacement and toepad aspect ratio) against the natural logarithm of SVL and used the residuals to model size relative traits. Then, we used mixed effect models, using the package *lme4* (Bates et al. 2015) with each size-relative trait as the response variable, hurricane date as a factor (−7 months, 16 months, and 27 months) and habitat (urban or forest) as fixed effects, and site as a random effect.

In each model, we considered hurricane effects by evaluating the estimated marginal means for each factor combination and its standard error, using the package R *emmeans* (Lenth et al. 2019), where we contrasted both of the post-hurricane populations to the pre-hurricane populations (+16 month in contrast to −7 month and +27 month in contrast to −7 month). With this approach, negative *t* values represent trait values, which decreased in post-hurricane populations, whereas positive *t* values represent an increase in trait values following the hurricanes. We adjusted our *P*-values for multiple testing using the false discovery rate method (Benjamini and Hochberg 1995). The effect of site was modeled as random due to unbalanced sampling of sites (see sampling methods). For each trait, we contrasted several models: A model that included the interaction term for hurricane date and habitat; a model without the interaction term; and, finally, if the additive model was overfitted, we ran an additional model only including hurricane date. Models were determined to be overfitted using the function *lmer*, which returns a parameter that evaluates whether the variance of one or more linear effect combinations are close to zero. Mixed effect models with zero or near zero variance are thought to be overfitted due to poor power. Following this approach, a significant effect of hurricane would indicate a shift in toe and toepad morphology between pre- and post-hurricane populations. A significant effect of habitat would indicate morphological differences between urban and forest sites. A significant interaction effect between hurricane and habitat would indicate specific morphological shifts in response to the hurricane within urban and forest sites. We used the AIC criterion to select the best fitting model and verified, using ANOVA, that models were statistically different. If we found that the additive and interactive models were non-differentiated, we chose the simpler model without the interaction term.

### Post-hurricane toe shape associated with clinging performance

Finally, we also explored toepad morphology-performance relationships. We only gathered performance data from our two post-hurricane observation dates (January and December 2019), from all four sites (Table 1). Since our clinging performance is a measurement of the summed forces generated by all four feet simultaneously, we could not directly compare the shape of any specific toe with its specific performance capabilities. Alternatively, to gain a general understanding of how toepad shape may correlate with performance, we correlated front toe shape and performance and rear toe pad shape and performance independently using Procrustes ANOVAs and Partial Least Square (PLS) analyses. Both of these approaches find an axis of correlation (Procrustes ANOVA) or covariation (PLS) between our geometric morphometric dataset and maximum cling force. We subsequently evaluated models correlating performance using linear measurements of toe and toepad morphology, to assess specific morphological features correlated with performance. While geometric morphometric analyses control for size during the alignment step, we also controlled for size in subsequent analyses. First, we used as a response in the models a measure of toepad area independent cling forces by using the residuals of the linear relationship between maximum cling force (N) and toepad area. Then, we used Analysis of Covariance (ANCOVA) to explore the correlations of residual cling forces with toepad aspect ratio, inter-lamellae height, lamellae displacement, toe length, and toepad area (covariate). We selected toepad area as our covariate, because this trait is significantly positively correlated with cling performance (Irschick et al. 1996), allowing us to evaluate other measurements of toe pad shape and organization. Since we expect the biomechanical principles connecting morphology and performance to be consistent across habitat types, we focused only on morphology associations with clinging performance in our analyses, not including habitat category or observation date.

## Results

### Toe shape in response to hurricanes

We first evaluated front and rear toe and toepad shape using Procrustes ANOVA. We then used a PCA to visualize the main axes of shape variation in our aligned datasets.

**Fig. 2:**
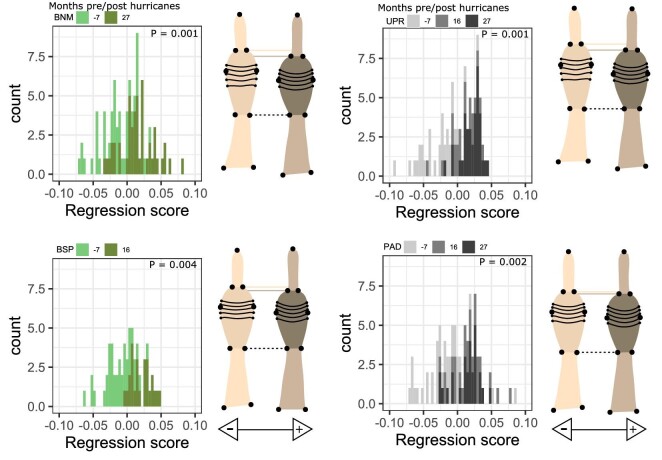
Front toe shape trajectories derived from Procrustes ANOVA correlating shape and observation date for each site. Colors correspond to forest habitats in green and urban habitats in grays, with darker shading representing later time points relative to Hurricane Maria. These include our sampling 7 months before the hurricanes, 16 months after the hurricanes, and 27 months after the hurricanes. The X axes correspond to the regression score of the ANOVA model. The regression scores represent the shape divergence from the predicted values generated by Procrustes ANOVA. Minimum (cream) and maximum (brown) regression score toe shape projections are presented side by side to the right of each plot for easy visual comparisons, as depicted by the black arrows beneath each pair of projections. The dotted black line highlights the base of both projections’ toepad. The dotted cream and brown lines highlight the distal end of the toepad for each projection.

**Fig. 3:**
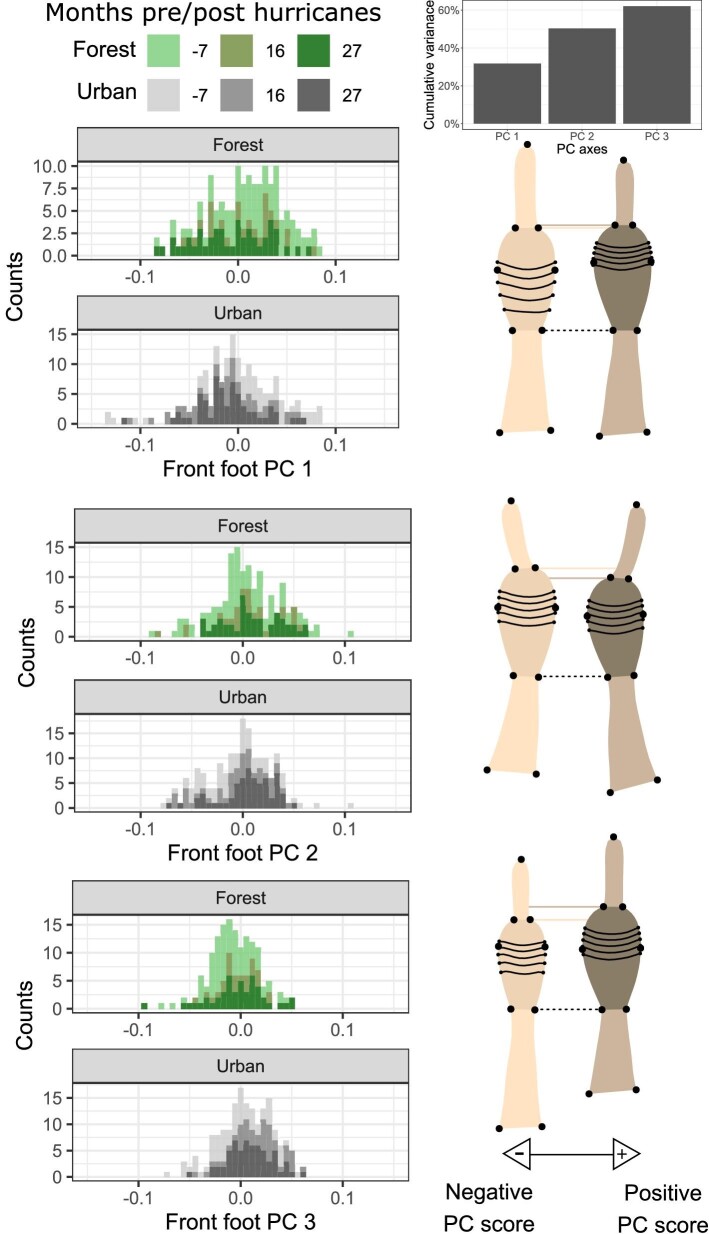
Front foot histograms of principal components one through three. Forest and urban individuals (green or gray histograms) are plotted separately along each PC axis, with darker shades representing later observations. Minimum (cream) and maximum (brown) shape projections illustrate morphological differentiation along each axis. The dotted black line highlights the base of both projections’ toepad. The dotted cream and brown lines highlight the distal end of the toepad for each projection.

*Fore foot—*We found that front toe shape changed significantly with time at all four sites evaluated separately (Procrustes ANOVA all *P* values < 0.01; Table 2). Following the hurricane, within-site shape projections (Fig. 2) show that individuals with positive regressions scores had toe pads that made up less of their toes’ overall length, resulting in shorter toepads. The histograms of all four sites are skewed toward more positive values in post-hurricane populations (Fig. 2). Principal components one through three captured 62% of the shape variation in our front foot dataset (Fig. 3). Individuals orientated toward high PC1 scores had more distally placed lamellae 6–11 and these lamellae appear to be closer together (reduced inter-lamellae spacing), and the distal toe segment (the distance from the distal end of the pad to the claw) appears longer. PC2 captured variation with respect to toe bending and toe/toepad proportions, which has been observed previously in geometric morphometric analysis of rear toepad shape (Howell et al. 2022), with high PC2 values representing toes whose overall length is comprised relatively less toepad and longer proximal toe segments. Similarly, PC3 captured variation in toe proportions, with positive PC3 values capturing toes consisting of proportionally more toepad with relatively wider pads and shorter proximal toe segments. Lamellae 6–11 are also more distally located among individuals with negative PC3 scores (Fig. 3).

**Table 2:** Site-specific Procrustes ANOVA for front and rear feet toe shape variation with hurricane context (7 months before, 16 and 27 months after Hurricane Maria).

Site	Habitat	Foot	F and P values
BSP	Forest	Front	*F*_1/62 _= 3.533; *P* = 0.004
BNM	Forest	Front	*F*_1/84_ = 5.235; *P* = 0.001
UPR	Urban	Front	*F*_1/84_ = 7.955; *P* = 0.001
PAD	Urban	Front	*F*_1/87_ = 4.313; *P* = 0.004
BSP	Forest	Rear	*F*_1/61 _= 5.235; *P* = 0.001
BNM	Forest	Rear	*F*_1/86_ = 9.185; *P* = 0.001
UPR	Urban	Rear	*F*_1/74_ = 7.123; *P* = 0.001
PAD	Urban	Rear	*F*_1/73_ = 9.537; *P* = 0.001

*Note*: Degrees of freedom are presented as subscripts of the *F* value and the *P* value.

We considered overall changes in body size before and after the hurricanes. Similar to previous studies (Donihue et al. 2018; Dufour et al. 2019; Rabe et al. 2020; Avilés-Rodríguez et al. 2021; Simon et al. 2023), we found significant changes in SVL attributed to both the hurricane and habitat (Fig. S1). Thus, to isolate the effect of body size, we regressed our linear trait measurements against the natural logarithm of body size (see Methods).

**Fig. 4:**
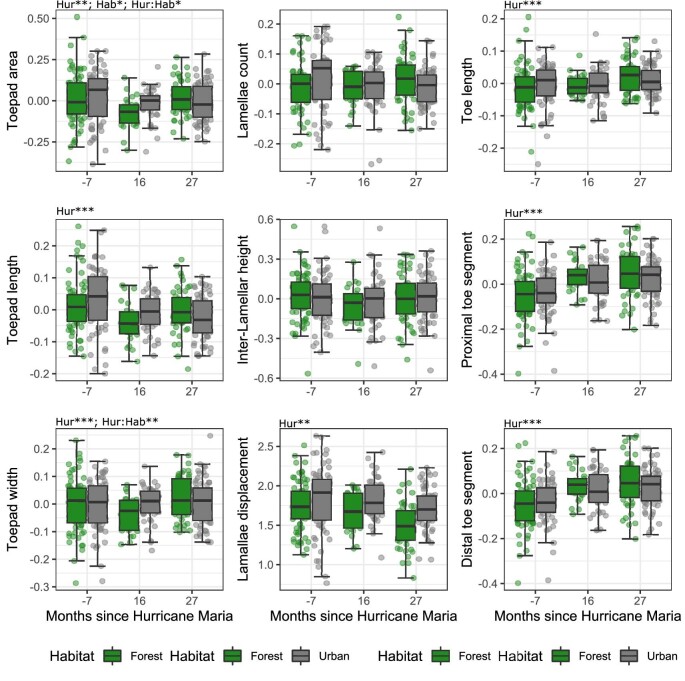
Univariate front toe morphology relative SVL for forest and urban populations of *Anolis cristatellus* before and after Hurricane Maria. We obtained the residuals of linear morphology regressed against SVL, except for traits measured as ratios (see Methods). Then, we subtracted the site mean to each observation to match mixed effect models where site variation is treated as a random effect. As such, the above plot is a representation of the variation in our groups and not an exact representation of our statistical models. Lamellae displacement refers to how distally placed the center of mass of lamellae 6–11 are on the toepad. The text above plots (e.g., Hur, Hab, and Hur: Hab) correspond to whether the effects of hurricane context, ecological context (i.e., urban versus forest), or their interaction were significant. Non-differentiated traits were left blank. Stars correspond to a significant threshold of “*” 0.05, “**” 0.01, or “***” 0.001.

*Front feet linear measurements—*Mixed effect models of univariate traits largely reiterated our previous Procrustes ANOVA shape analyses. Forest lizards sampled at 16 months after the hurricane had smaller relative **toepad areas (area relative to SVL)** than pre-hurricane forest populations (Fig. 4; 16 month after: *T* = −2.921; *P* = 0.010; 27 month after: *T* = 0.642, *P* = 0.797). In contrast, relative toepad area did not significantly change in urban populations (16 month after: *T* = −1.855, *P* = 0.153; 27 month after: *T* = −1.435, *P* = 0.324). Our model structure for **toepad lengths relative to SVL** resulted in an overfitted model, thus we verified the main effect of hurricane context in a separate model without including habitat (see Supplementary Table 1 for more details). We found that post-hurricane **toepads lengths (lengths relative to SVL)** were shorter at both 16 and 27 months after the hurricane (16 month after: *T* = −3.435. *P* = 0.002; 27 month after: *T* = −3.559. *P* = 0.001). **Toepad widths (widths relative to SVL)** had marginally significant changes post-hurricane in forest (16 month after: *T* = −2.16, *P* = 0.080; 27 month after: *T* = 2.207, *P* = 0.073) but not urban populations (16 month after: *T* = 0.573, *P* = 0.834; 27 month after: *T* = 1.138, P = 0.491). In forest populations, relative toepad width first decreased at 16 months and then increased towards pre-hurricane values at 27 months after the hurricane. We found no significant effect of either hurricane or habitat on **lamellae counts** (16 month after: *T* = −0.712, *P* = 0.756; 27 month after: *T* = −1.247, P = 0.427) or relative **inter-lamellae height** (16 month after: *T* = −1.602, *P* = 0.246; 27 month after: *T* = −0.232, *P* = 0.970). Our models of **lamellae displacement** were overfitted, thus we removed habitat as a fixed effect. We found that post-hurricane lizards had lamella shifted toward the proximal end of the toepad (16 month after: *T* = −3.507, *P* = 0.001, 27 month after: *T* = −6.711, *P* < 0.001). We found parallel changes in **toe lengths (relative to SVL)** for forest and urban lizards. Populations at 16 months post-hurricane did not differ in toe lengths, but populations sampled at 27 months had relatively longer toes (16 month after: *T* = 0.283, *P* = 0.969; 27 month after: *T* = 3.414, *P* = 0.002). For **proximal and distal toe segments lengths** relative to SVL, we found parallel increases in relative lengths in response to the hurricane for urban and forest populations. Post-hurricane populations had significantly longer relative **proximal toe segment lengths** (16 month after: *T* = 4.252, *P* < 0.001; 27 month after: *T* = 6.336, *P* < 0.001). Similarly, post-hurricane populations had significantly longer relative **distal toe segment lengths** (16 month after: *T* = 2.527, *P* = 0.032; 27 month after: *T* = 5.291, *P* < 0.001).

**Fig. 5:**
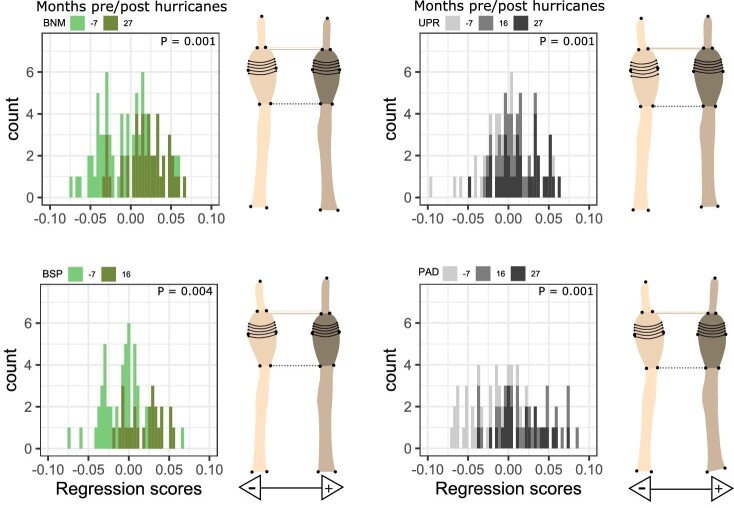
Rear toe shape trajectories derived from our Procrustes ANOVA analysis for each site correlating shape with observation date relative to the hurricanes. Colors correspond to forest habitats in green and urban habitats in grays with darker shading showing time since Hurricane Maria. The X axes correspond to the regression score of the ANOVA model. The regression scores represent the shape divergence from the predicted values generated by Procrustes ANOVA. Minimum (cream) and maximum (brown) regression score toe shape projections are presented side by side to the right of each plot for easy visual comparisons, as depicted by the black arrows beneath each pair of projections. The dotted black line highlights the base of both projections’ toepad. The dotted cream and brown lines highlight the distal end of the toepad for each projection.

**Fig. 6:**
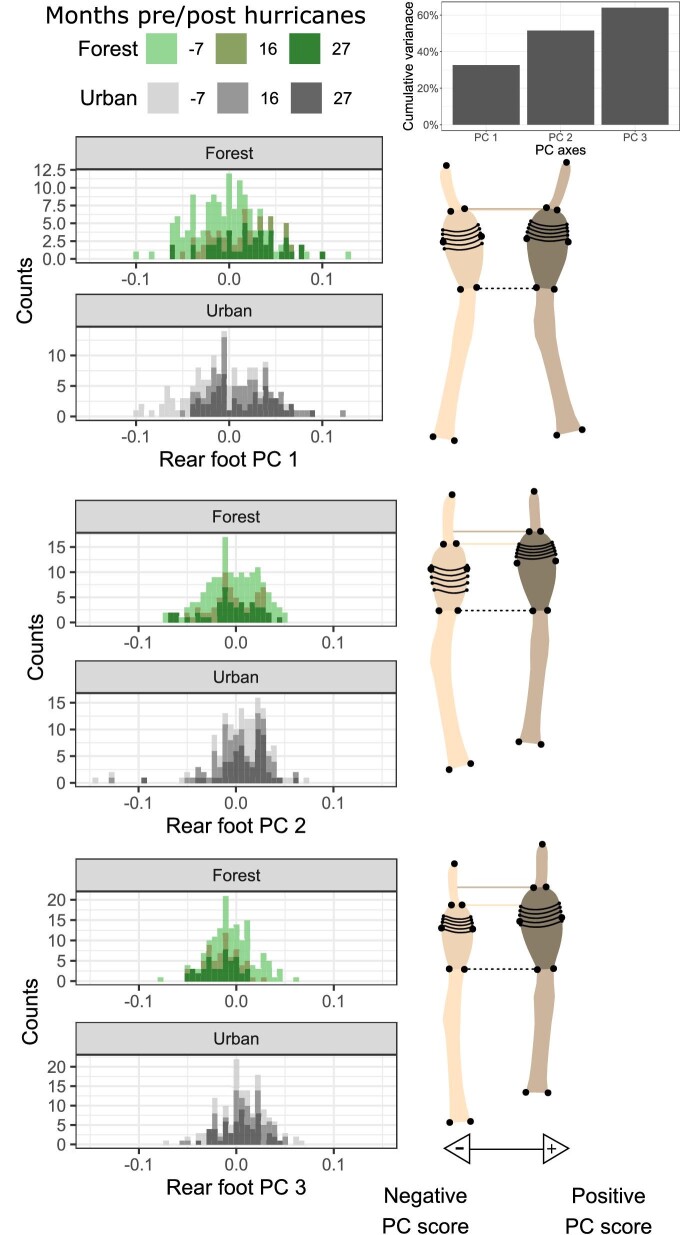
Rear foot histograms of principal components one through three. Forest and urban individuals (green or gray histograms) are plotted separately along each PC axis, with darker shades representing later observations. Minimum (cream) and maximum (brown) shape projections illustrate morphological differentiation along each axis. The dotted black line highlights the base of both projections’ toepad. The dotted cream and brown lines highlight the distal end of the toepad for each projection.

*Hind Foot—*Considering hind toe shape through time within each site separately, we found that rear toe shape changed significantly with hurricane context for all four sites (Procrustes ANOVA all *P* values < 0.01; Table 2). Shape variation along the regression scores suggests more subtle changes in shape in contrast to front toes (Fig. 5). Individuals with positive regression scores had shorter toepads. Principal components one through three accounted for a cumulative variation of 64.0% of the data (Fig. 6). PC1 captured variation with respect to toe bending, which is an artifact of data collection, and lamellae location, similar to Howell et al. (2022). Individuals higher on PC1 have distally displaced lamellae. Lizards with greater PC2 scores had elongated toepads with a distal placement of more tightly spaced focal lamellae and shorter proximal toe segments. PC3 corresponded to variation in toepad shape, where individuals with positive PC3 scores had relatively enlarged toepads and shorter proximal toe segments.

**Fig. 7:**
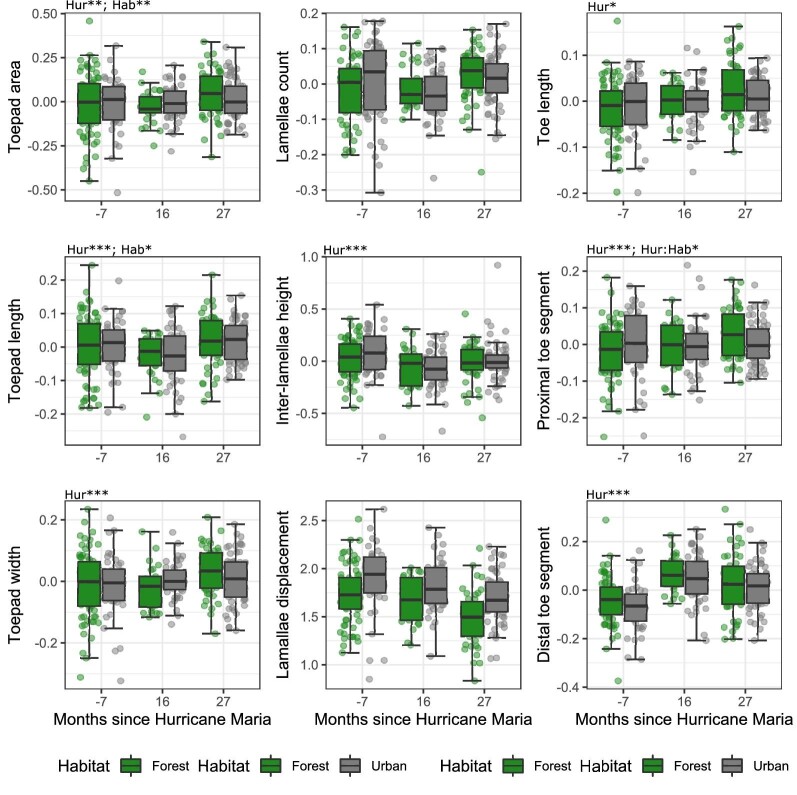
Univariate rear toe morphology relative to SVL of forest and urban populations of *Anolis cristatellus* before and after Hurricane Maria. We obtained the residuals of linear morphology regressed against SVL, except for traits measured as ratios (see Methods). Then, we subtracted the site mean to each observation to match mixed effect models where site variation is treated as a random effect. As such, the above plot is a representation of the variation in our groups and not an exact representation of our statistical models. Lamellae displacement refers to how distally placed the center of mass of lamellae 6–11 are on the toepad. The text above plots (e.g., Hur, Hab, and Hur: Hab) correspond to whether the effects of hurricane context, ecological context (i.e., urban versus forest), or their interaction were significant. Non-differentiated traits were left blank. Stars correspond to a significant threshold of “*” 0.05, “**” 0.01, or “***” 0.001.

*Rear foot linear trait analyses—*We found a series of significant morphological changes post-hurricane (Fig. 7). We found parallel changes in relative **toepad area** (area relative to SVL) post-hurricane in urban and forest populations. Toepad areas were larger at 27 months but not at 16 months after the hurricane (16 month after: *T* = −0.170, P = 0.984; 27 month after: *T* = 2.913, *P* = 0.011). Changes in relative **toepad length** (lengths relative to SVL) were also parallel between habitat types, with initially shorter toepads 16 months after the hurricane and then longer at 27 months after (16 month after: *T* = −2.544, *P* = 0.031; 27 month after: *T* = 2.351, *P* = 0.051). Our model structure for relative **toepad width** (width relative to SVL) resulted in an overfitted model, thus we verified the main effect of hurricane context in a separate model without including habitat (see Supplementary Table 1 for more details). We found that post-hurricane lizards had wider toepads at 27 after but not at 16 months after (16 month after: *T* = 0.937, *P* = 0.6176; 27 month after: *T* = 3.589, *P* = 0.001). We found no significant effect of either hurricane or habitat on **lamellae counts** (relative to SVL; 16 month after: *T* = −0.987, *P* = 0587, 27 month after: *T* = 1.262, *P* = 0.418). Our model structure was overfitted for **inter-lamellar height** (height relative to SVL), thus we fit a simpler model without the effect of habitat. We found that post- hurricane lizards had more tightly spaced lamellae at 16 months but not at 27 months after the hurricane (16 month after: *T* = −3.670, *P* < 0.001; 27 month after: *T* = −1.557, *P* = 0.266). Our model of **lamellae displacement** was overfitted, thus we fit a simpler model including only the effect of the hurricanes. We found no significant shifts in lamellae displacement associated with the hurricanes (16 month after: *T* = −0.540, *P* = 0.856, 27 month after: *T* = 1.817, *P* = 0.167). Our models of **toe lengths** (lengths relative to SVL) were overfitted even after removing habitat as a fixed effect. We verified the main effect of the hurricane by evaluating a simplified model with the absolute **toe lengths**, the fixed effect of the hurricane and the random effect of SVL. Post-hurricane populations had greater **toe lengths** at 16 months but not at 27 months after the Hurricane (16 month after: *T* = 2.623, *P* = 0.023; 27 month after: *T* = 0.623, *P* = 0.808). For **proximal toe segment lengths** (lengths relative to SVL), we found that forest populations had longer lengths 27 months after the hurricane (16 month after: *T* = 0.059, *P* = 0.998; 27 month after: *T* = 3.625; *P* < 0.001). Urban populations had similar **proximal toe segments** following the hurricane (16 month after: *T* = −0.066, *P* = 0.998; 27 month after: *T* = 0.146; *P* = 0.988). Post-hurricane populations had significantly longer **distal toe segment lengths** irrespective of the habitat (16 month after: *T* = 7.066, *P* < 0.001; 27 month after: *T* = 4.441; *P* < 0.001).

### Post-hurricane toe shape associated with clinging force

**Fig. 8:**
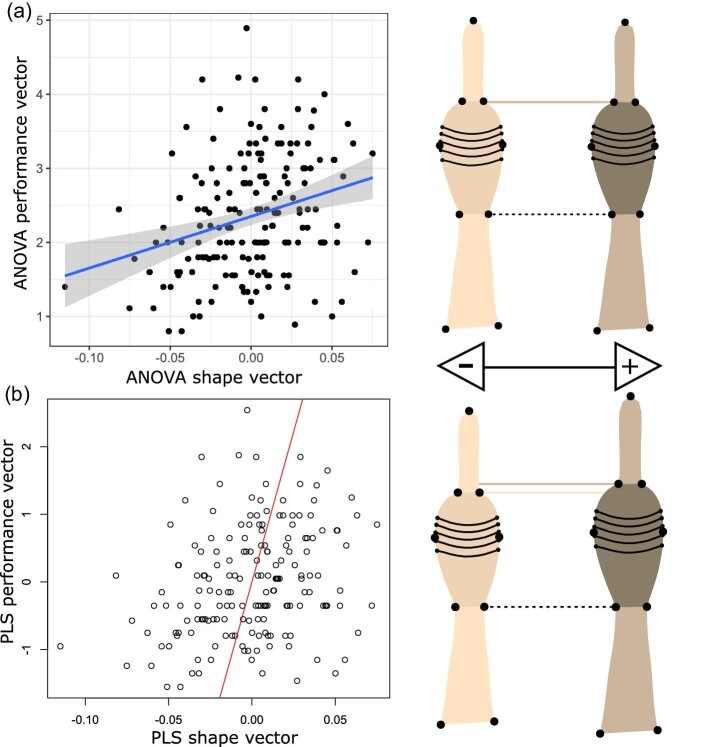
Correlations of maximum cling force (N) with predicted front toe shape using (A) Procrustes ANOVA and (B) PLS analyses. Minimum (cream) and maximum (brown) shape projections illustrate morphological differentiation along each the *X*-axis of each model. The dotted black line highlights the base of both projections’ toepad. The dotted cream and brown lines highlight the distal end of the toepad for each projection.

**Fig. 9:**
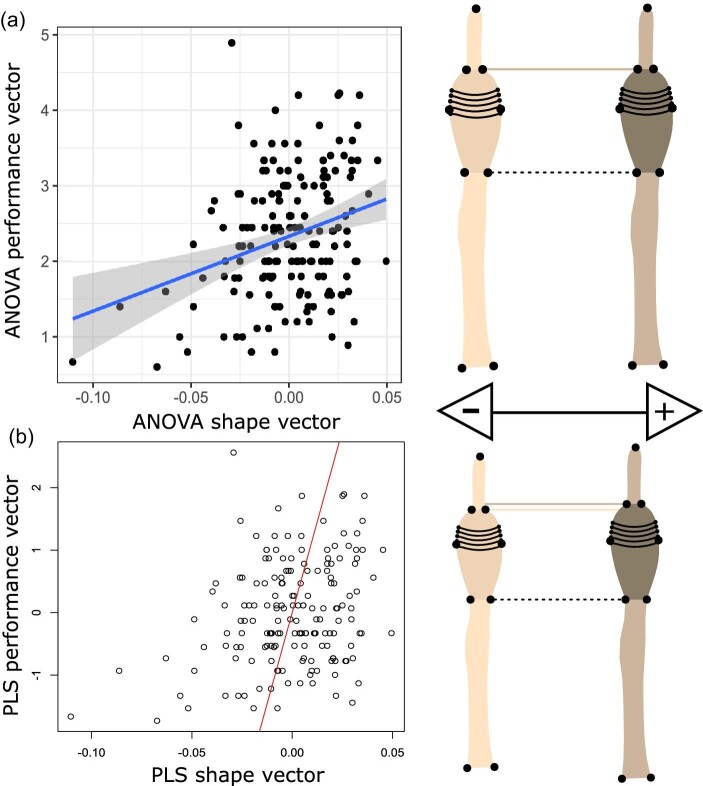
Correlations of maximum cling force (N) with predicted rear toe shape using (A) Procrustes ANOVA and (B) PLS analyses. Minimum (cream) and maximum (brown) shape projections illustrate morphological differentiation along each the *X*-axis of each model. The dotted black line highlights the base of both projections’ toepad. The dotted cream and brown lines highlight the distal end of the toepad for each projection.

Both front and rear toe shape varied significantly with maximum clinging force (Procrustes ANOVA; **front**: *F*_1/176_ = 3.179, *P* = 0.005; **rear**: *F* = _1/171_ = 2.238, *P* = 0.036). Using PLS analyses, we found a significant covariance between toe and toepad shape with cling force relative to toepad areas (Figs. 8 and 9; front feet PLS: *Z* = 2.932, *P* = 0.001; rear feet PLS: *Z* = 2.339, *P* = 0.001). ANCOVA results of cling force explained by univariate toepad morphology when controlling for toepad area did not recapitulate these results. Instead, we found that toepad area, and toe length largely informed performance. In front feet, we found that the overall model was significant (*F*_6/170_ = 5.704, *P* < 0.001), but only toepad length and toepad area had a significant effect on performance (*F*_1/170 _= 30.317; *P*, 0.001 & *F*_1/170 _= 23.910; *P* < 0.001; Table 3). Similarly, in rear feet, we found that overall model was significant (*F*_6/165 _= 5.528, *P* < 0.001), but only toepad length, lamellae counts, and toepad area were correlated with performance (Table 3).

**Table 3:** Results from the ANCOVA models testing for the effects of toepad aspect ratio, inter-lamellae height, lamellae displacement, toe length, and toepad area (covariate) on residual cling force (i.e., residuals of maximum cling force (N) relative to toepad area).

	Toepad aspect ratio	Inter-lamellae height	Lamellae displacement	Lamellae count	Toe length	Toepad area (covariate)	Overall model
Front feet	*F*_1/170 _= 1.066 *P* = 0.303	*F*_1/170 _= 0.033 *P* = 0.856	*F*_1/170 _= 0.764 *P* = 0.383	*F*_1/170 _= 0.093 *P* = 0.760	*F*_1/170 _= 30.317 *P* < 0.001	*F*_1/170 _= 23.910 *P* < 0.001	*F*_6/170 _= 5.704 *P* < 0.001
Rear feet	*F*_1/165 _= 0.000 *P* = 0.980	*F*_1/165 _= 0.018 *P* = 0.892	*F*_1/165 _= 0.054 *P* = 0.816	*F*_1/165 _= 4.423 *P* = 0.370	*F*_1/165 _= 22.860 *P* < 0.001	*F*_1/165 _= 27.844 *P* < 0.001	*F*_6/165 _= 5.528 *P* < 0.001

*Note*: We evaluated separate models for the front and rear feet.

## Discussion

Hurricanes are powerful storms that can drive changes in habitats and species (Schowalter 1994; Spiller and Schoener 2007). Ongoing climate change is projected to increase the frequency and severity of hurricanes (Zhao et al. 2009; Bender et al. 2010). Concomitantly, many species are experiencing variable degrees of anthropogenic disturbance such as urbanization. Nevertheless, how these two forms of disturbance may compound one another, or to what degree highly urbanized populations respond to severe weather events is largely unknown. Here, we evaluate whether urban populations of lizards in Puerto Rico responded differently than forest counterparts following 2017’s Hurricanes Maria and Irma. We found that toe and toepad shape changes, following the hurricanes, varied in magnitude and directionality, depending on the ecological context (urban versus forest), and between the front and rear feet.

### Hurricane responses across urban and forest habitats for front and rear toes

We predicted a significant interaction between habitat type and the effect of hurricanes based on the increased toepad size of anoles in urban populations (Winchell et al. 2018; Howell et al. 2022). For instance, prior research suggests that urbanization and hurricanes may both generate significant positive selection for toepad area (Winchell et al. 2016; Donihue et al. 2018). In that case, urban populations may respond differently or not at all to hurricanes, given that urban phenotypes are similar to phenotypes associated with hurricane tolerance. Indeed, we found that while post-hurricanes forest populations experienced a *reduction* in front toe toepad area and width (area and width relative to SVL), urban front toes remained largely the same before and after the hurricanes (Fig. 4). In contrast, we found that both urban and forest populations experienced *increases* in rear toepad area, toepad length, and toepad width (all traits relative to SVL; Fig. 7). Interestingly, rear toe shape results recapitulate previous findings of larger toepads areas associated with hurricanes in non-urban populations (Donihue et al. 2018, 2020; Simon et al. 2023). In contrast, our results considering front toes largely recapitulate prior findings, on other *A. cristatellus* populations, of smaller toe morphology in forest, but not urban, environments (Avilés-Rodríguez et al. 2021). The non-parallel morphological shifts between urban and forest habitats may be due to toepad selection imposed by the higher frequency of broader and smoother surfaces in urban habitats (Winchell et al. 2018; Falvey et al. 2020; Avilés-Rodríguez et al. 2021).

Our finding of variable trait responses of front and rear toes likely reflects a diverging biomechanical role of these phenotypic characteristics in the context of hurricanes and urban substrate use. For example, laboratory wind trials show that front feet and limbs maintain surface contact longer than rear feet under unidirectional wind exposure (Donihue et al. 2018; Debaere et al. 2021). On vertical cylindrical perches, the lizard's body is partially shielded from the wind by the perch, and grasping strengths increase when longer limbs allow a fuller grasp of the perch and by the interlocking of front toes (Kolbe 2015; Debaere et al. 2021). In contrast, rear feet lose contact with the perch first, with longer hindlimbs increasing the likelihood of dislodgement due to higher drag forces on the exposed hindlimbs (see Donihue et al. 2018; Debaere et al. 2021 for videos and images). Contrasting front and rear toe and limb morphology patterns have been reported in the context of urbanization (Winchell et al. 2018). For instance, longer forelimbs increase the likelihood of slips and falls when running on smooth broad vertical surfaces because they push the center of mass away from the perch (Winchell et al. 2018). Longer rear limbs and larger rear toepad areas decreased the likelihood of slips and falls, providing greater stability during vertical locomotion. The selective effect of forest and urban environments during hurricanes may interact in complex ways, generating different evolutionary responses in front and rear limbs. Characterizing the interaction of front and rear limb biomechanics in the context of hurricanes and urban substrate use will require further laboratory experimentation, simulating the full range of possibilities of wind exposure (i.e., lizards perching on artificial and natural substrates under high wind conditions).

It is also important to recognize that other factors could be contributing to long-term shifts in anole morphology. For example, increases in toepad area are associated with shifts in perching heights (Kamath et al. 2020). Additionally, changes in temperature during incubation have been linked with small changes in SVL, body mass, and body condition (Hall and Warner 2018; Pruett and Warner 2021). The reproductive peak of our focal species occurs during the summer months (Gorman and Licht 1974; Otero, Huey, and Gorman 2015), which could impact the local abundance of larger males within our sites. Our sampling, primarily dictated by access to sites rather than intentionally designed, occurred at nearly yearly intervals during the dry, non-reproductive season. Thus, by chance, we have reduced some stochasticity arising from seasonal shifts and reproductive peaks. Despite the many correlates with anole morphology, recent studies have shown increasing toepad areas on islands that experience greater hurricane disturbances (Donihue et al. 2020). Thus, hurricanes are likely an important factor underlying short- and long-term changes in toepad morphology for anole lizards.

### Toe and toepad shape contributions to clinging performance

Previous studies have documented a strong correlation between toepad area and maximum clinging forces in *Anolis* and other pad-bearing lizards (Irschick et al. 1996; Elstrott and Irschick 2004; Gilman et al. 2015). We expected to find one or more dimensions of morphology beyond toepad area associated with performance. Our geometric morphometric analyses (Figs. 8 and 9) revealed a significant axis of shape correlated with performance. However, our univariate analyses using toepad area as a covariate indicated that toepad area and toe length are strongest predictors of clinging performance. This suggests that total pad area and not shape (width versus lengths) are the main predictors of maximum adhesive forces. We were not able to directly contrast pre- and post-hurricane clinging performance. The morphological shifts following the hurricane could be linked with both increases or decreases in clinging performance. Specifically, we saw *decreases* in the front toepad area, and *increases* in both rear and front toepad area and toe lengths following the hurricanes. However, external toepad morphology alone might not be a predictor of cling force following a hurricane as was reported in Dufour et al. (2019). We suggest further biomechanical research is needed to better evaluate the interactions between toe lengths and toepad areas for producing maximum adhesion.

Our ability to generalize our clinging performance findings is confounded by the high variability of methods employed to measure clinging performance across studies (Supplementary Table 2). Previous studies have evaluated lizard clinging performance using one hind foot (Losos, Walton, and Bennett 1993), one front foot (Dufour et al. 2019; Wright et al. 2021), both front feet (Bloch and Irschick 2005), all four feet (Zani 2000; Pillai et al. 2020), as well as vertical or horizontal surfaces. Clinging performance has also been defined as the ratio of forces produced by a single rear toe (Hagey et al. 2016). We opted to measure whole animal performance (all four feet) vertically to better simulate the behavior of animals clinging to vertical surfaces (Winchell et al. 2016, 2018; Falvey et al. 2020). Nevertheless, we found substantial variation between front and rear feet morphology, indicating that future studies should consider the biomechanical role of these traits independently and how they might impact their chosen methodology and interpretation.

## Conclusion

Studying the impacts of hurricanes and other extreme weather events is imperative, especially when habitats are concurrently affected by urbanization. We evaluated a known urban adapter *A. cristatellus* (Winchell et al. 2016, 2018), and found major differences in the phenotypic responses to hurricanes between urban and forest populations. Other studies have documented phenotypic changes in anoles following a major hurricane (Donihue et al. 2018, 2020; Dufour et al. 2019; Rabe et al. 2020; Avilés-Rodríguez et al. 2021; Simon et al. 2023) with varying responses in the toepad morphology. However, no study prior to Howell et al. (2022) had used geometric morphometric approaches to evaluate anole toepad morphology. We found contrasting morphological changes in the front and rear feet of urban and forest populations. Urbanization was a significant axis for divergent variation following the hurricane for front toes (Fig. 4), but not for rear toes (Fig. 7). This suggests that urban populations can experience divergent responses to extreme events, but in our focal species, this differed between the front and rear feet. Thus, both front and rear toes should be considered when investigating morphological shifts related to anole clinging performances. Together, our results indicate that future research aiming to understand how populations are affected by urbanization also need to consider the impact of extreme climate events.

## Supplementary Material

obad025_Supplemental_File

## Data Availability

Data are available on the Dryad Digital Repository (https://doi.org/10.5061/dryad.vmcvdncxj).
