# Supplementary material for: Geometric Morphometric Assessment of Toe Shape in Forest and Urban Lizards Following Hurricane Disturbances
Source: Integr Org Biol. 2023 Jul 12;5(1):obad025. doi: 10.1093/iob/obad025 (PMC10384016; doi:10.1093/iob/obad025)
Supplement: obad025_Supplemental_File [file obad025_supplemental_file.docx]

#### Supplements


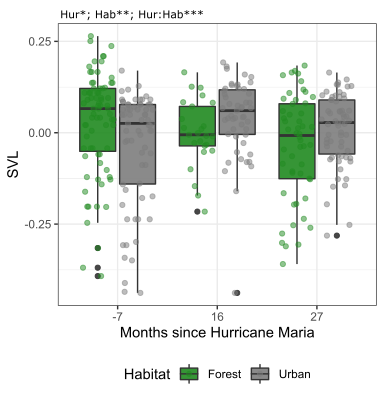


Figure S1: Changes in body size (i.e., SVL) of forest and urban populations of Anolis cristatellus before and after Hurricane Maria. We subtracted the site mean to each observation to match mixed effect models where site variation was treated as a random effect. Hur, Hab and Hur:Hab above the plot denote whether the effects of hurricane context, ecological context (i.e., urban versus forest) or their interaction were significant. Stars correspond to a significant threshold of ‘*’ 0.05, ‘**’ 0.01 or ‘***’ 0.001. Our findings of hurricane-induced changes in body size recapitulate previous findings of increases in lizard body sizes after the hurricane in some populations (Dufour et al, 2019) but also decreases in other populations (Donihue et al 2018, Aviles-Rodriguez et al 2021). Specifically, we found that forest populations were significantly smaller at 27 months post hurricane, but not at 16 months after the hurricane when compared to the body lengths of forest individuals 7 months before the hurricanes (16 mo. after: T = 0.107. P = 0.994; 27 mo. after: T = -3.631. P = 0.001). In contrast, urban populations had larger bodies at 16 months, but not at 27 months after the hurricane (16 mo. after: T = 3.253. P = 0.003; 27 mo. after: T = 1.8525. P = 0.163).

Supplementary Table 1: Contrast of mixed effect model fits. Our models evaluated traits values relative to SVL (except for lamellae counts and lamellae displacement) against the fixed effects of the hurricanes (-7, 16, 27 months before or after the storms) and habitat (urban versus forest) with the random effects of sites (BSP, BNM, UPR, PAD). We first evaluated a model including the interaction term of our fixed effects (hurricane X habitat) and a model without the interaction term (hurricane + habitat). We selected the model with the best fit based on the AIC criterion and using ANOVA test. When the interactive and additive model were not significantly differentiated we reported the results of the simpler additive model. In cases when the interactive and additive were overfitted, we evaluated a separate model without the effect of habitat. In the rare case of this simplified model also overfitting we evaluated the main effect of hurricane on the absolute trait value with SVL as a random effect. Bold lettering denotes model contrast reported in the manuscript for each trait.

Interactive model: Trait relative to SVL ~ Hurricane X Habitat + (1|Site)

Additive model: Trait relative to SVL ~ Hurricane + Habitat + (1|Site)

Hurricane only model: Trait relative to SVL ~ Hurricane + (1|Site)

Simplified hurricane only model :Absolute trait ~ Hurricane + (1|SVL)

| Trait | Model | k | Bo | B16mo | B27mo | Burb | B16mo X  urb. | B27mo. X  urb. | log(L) | AIC |
| --- | --- | --- | --- | --- | --- | --- | --- | --- | --- | --- |
| **SVL** | **Interactive** | **8** | **6.287** | **0.021** | **-0.614** | **0.047** | **0.442** | **0.866** | **-381.972** | **779.945** |
| SVL | Additive | 6 | 6.104 | 0.213 | -0.013 | 0.433 | - | - | -391.024 | 794.048 |
| **Fore toepad area** | **Interactive** | **8** | **-0.011** | **-0.010** | **0.020** | **0.057** | **0.052** | **-0.055** | **190.507** | **-365.015** |
| Fore toepad area | Additive | 6 | -0.008 | -0.063 | -0.011 | 0.048 | - | - | 187.698 | -363.396 |
| Fore toepad length | Interactive | 8 | 0.004 | -0.054 | -0.019 | 0.037 | 0.016 | -0.036 | 354.356 | -692.712 |
| Fore toepad length | Additive | 6 | 0.008 | -0.042 | -0.038 | 0.027 | - | - | 351.592 | -691.198 |
| **Fore toepad length** | **Hurricane only** | **5** | **0.022** | **-0.040** | **-0.039** | **-** | **-** | **-** | **348.241** | **-686.482** |
| **Fore toepad width** | **Interactive** | **8** | **-0.013** | **-0.046** | **0.043** | **0.010** | **0.056** | **-0.026** | **332.746** | **-649.492** |
| Fore toepad width | Additive | 6 | -0.015 | -0.011 | 0.028 | 0.013 | - | - | 328.112 | -644.22 |
| Fore lamellae counts | Interactive | 8 | 12.905 | -0.174 | 0.122 | 0.856 | -0.021 | -0.477 | -479.356 | 974.711 |
| **Fore lamellae counts** | **Additive** | **6** | **13.013** | **-0.128** | **0.678** | **-** | **-** | **-** | **-480.085** | **972.170** |
| Fore inter-lamellar height | Interactive | 8 | -0.006 | -0.075 | -0.016 | 0.033 | 0.052 | 0.023 | 89.890 | -163.779 |
| **Fore inter-lamellar height** | **Additive** | **6** | **-0.015** | **-0.044** | **-0.006** | **0.053** | **-** | **-** | **89.436** | **-166.871** |
| Fore lamellae displacement | Interactive | 8 | 0.240 | -0.013 | -0.026 | 0.008 | -0.003 | -0.003 | 720.693 | -1425.387 |
| Fore lamellae displacement | Additive | 5 | 0.241 | -0.014 | -0.024 | 0.007 | - | - | 720.553 | -1429.107 |
| **Fore lamellae displacement** | **Hurricane only** | **5** | **0.244** | **-0.014** | **-0.023** | **-** | **-** | **-** | **718.099** | **-1426.197** |
| Fore toe length | Interactive | 8 | -0.014 | -0.001 | 0.043 | 0.007 | 0.001 | -0.028 | 449.967 | -883.933 |
| **Fore toe length** | **Additive** | **6** | **-0.009** | **0.002** | **0.027** | **-0.002** | **-** | **-** | **448.018** | **-884.036** |
| Fore proximal toe length | Interactive | 8 | -0.060 | 0.063 | 0.120 | 0.030 | -0.011 | -0.059 | 285.069 | -554.139 |
| **Fore proximal toe length** | **Additive** | **6** | **-0.050** | **0.062** | **0.086** | **0.008** | **-** | **-** | **282.604** | **-553.208** |
| Fore distal toe length | Interactive | 8 | -0.060 | 0.063 | 0.120 | 0.030 | -0.011 | -0.059 | 285.069 | -554.139 |
| **Fore distal toe length** | **Additive** | **6** | **-0.050** | **0.062** | **0.086** | **0.007** | **-** | **-** | **282.604** | **-553.208** |
| Hind toepad area | Interactive | 8 | -0.055 | -0.043 | 0.089 | 0.069 | 0.055 | -0.048 | 164.644 | -313.288 |
| **Hind toepad area** | **Additive** | **6** | **-0.054** | **-0.003** | **0.060** | **0.065** | **-** | **-** | **162.189** | **-312.378** |
| Hind toepad length | Interactive | 8 | -0.022 | -0.050 | 0.040 | 0.039 | 0.024 | -0.018 | 316.007 | -616.015 |
| **Hind toepad length** | **Additive** | **6** | **-0.022** | **-0.032** | **0.0281** | **0.039** | **-** | **-** | **314.948** | **-617.835** |
| Hind toepad width | Interactive | 8 | -0.029 | -0.019 | 0.063 | 0.023 | 0.042 | -0.034 | 292.201 | -568.414 |
| Hind toepad width | Additive | 6 | -0.023 | 0.001 | 0.044 | 0.021 | - | - | 288.425 | -564.851 |
| **Hind toepad width** | **Hurricane only** | **5** | **-0.021** | **0.013** | **0.04** | **-** | **-** | **-** | **286.551** | **-563.098** |
| Hind Lamellae counts | Interactive | 8 | 15.600 | -0.182 | 0.614 | 1.050 | -0.140 | -0.581 | -547.923 | 1111.846 |
| **Hind lamellae counts** | **Additive** | **6** | **15.726** | **-0.210** | **0.226** | **0.798** | **-** | **-** | **-548.667** | **1109.334** |
| Hind inter-lamellar height | Interactive | 8 | 0.011 | -0.097 | -0.0357 | 0.086 | -0.056 | -0.40 | 68.0156 | -120.031 |
| Hind inter-lamellar height | Additive | 6 | 0.021 | -0.125 | -0.052 | 0.057 | - | - | 67.509 | -123.018 |
| **Hind inter-lamellar height** | **Hurricane only** | **5** | **0.045** | **-0.113** | **-0.045** | **-** | **-** | **-** | **64.661** | **119.332** |
| Hind lamellae displacement | Interactive | 8 | 0.189 | -0.004 | 0.002 | 0.007 | 0.004 | 0.008 | 732.472 | -1448.943 |
| Hind lamellae displacement | Additive | 6 | 0.187 | -0.002 | 0.006 | 0.011 | - | - | 731.313 | -1450.652 |
| **Hind lamellae displacement** | **Hurricane only** | **5** | **0.192** | **-0.002** | **0.005** | **-** | **-** | **-** | **727.712** | **-1445.424** |
| Hind toe length | Interactive | 8 | -0.021 | 0.014 | 0.045 | 0.013 | -0.002 | -0.026 | 421.714 | -827.428 |
| Hind toe length | Additive | 6 | -0.017 | 0.016 | 0.030 | 0.003 | - | - | 420.715 | -829.430 |
| Hind toe length | Hurricane only | 5 | -0.016 | 0.017 | 0.030 | - | - | - | 420.592 | -831.185 |
| **Hind toe length** | **Simplified hurricane** | **5** | **11.960** | **0.621** | **0.137** | **-** | **-** | **-** | **-427.301** | **954.600** |
| Hind proximal toe length | Interactive | 8 | -0.010 | -0.001 | 0.061 | 0.005 | 0.00006 | -0.052 | 338.443 | -660.886 |
| **Hind proximal toe length** | **Additive** | **6** | **-0.003** | **0.009** | **0.026** | **-0.015** | **-** | **-** | **335.003** | **-658.005** |
| Hind distal toe length | Interactive | 8 | -0.048 | 0.127 | 0.050 | -0.010 | -0.015 | 0.027 | 247.509 | -479.018 |
| **Hind distal toe length** | **Additive** | **6** | **-0.050** | **0.114** | **0.068** | **-0.004** | **-** | **-** | **246.808** | **-481.615** |

Supplementary Table 2: Preliminary summary of published methodology of clinging force trails of squamates. We did a google scholar search on the term “clinging performance” + “squamates” +
“Force” on December 8th 2022 and report the clinging performance trial methodology, focal feet and species evaluated in the pertinent 30 articles yielded within our query. We also supplemented our list by incorporating pertinent articles cited within our literature review. This is not meant to be an exhaustive search but rather an exercise to highlight the variation in the methodology for measuring cling force methods within a preliminary literature search. We also note that our search query yielded results largely biased towards squamates with padded toes.

| Citation | Method of force trial | Focal feet | Species |
| --- | --- | --- | --- |
| Autumn, K., Dittmore, A., Santos, D., Spenko, M. and Cutkosky, M., 2006. Frictional adhesion: a new angle on gecko attachment. *Journal of Experimental Biology*, *209*(18), pp.3569-3579. | “Setal array specimens were mounted on scanning electron microscope (SEM) stubs and evaluated with a custom two-axis mechanical tester. “ | Isolated setal array | Gekko gecko |
| Dufour, C. M. S., Donihue, C. M., Losos, J. B., & Herrel, A. (2019). Parallel increases in grip strength in two species of Anolis lizards after a major hurricane on Dominica. Journal of Zoology, jzo.12685. https://doi.org/10.1111/jzo.12685 | “The right fore-foot of the lizard was gently pressed downward on the slide and pulled across the surface with a constant speed for one minute (average of 10 clinging force measurements per minute.” | Right front foot | Anolis oculatus and Anolis cristatellus |
| Garner, A., Lopez, S., & Niewiarowski, P. H. (2017). Brown anole (Anolis segrei) adhesive forces remain unaffected by partial claw clipping). Acta Herpetologica, 12(2), 133–137. https://doi.org/10.13128/Acta_Herpetol-20572 | “Clinging experiments were completed using a custom designed, motorized rig that measured adhesive force. The first set of trials were con-ducted on a sheet of glass covered with an acetate sheet” | Front two feet | Anolis sagrei |
| Gilman, C. A. et. al, (2015). Geckos as Springs: Mechanics Explain Across-Species Scaling of Adhesion. *PLOS ONE*, *10*(9), e0134604. https://doi.org/10.1371/journal.pone.0134604 | “We measured maximum adhesive force (Fc) and system compliance (C) under shear loading  on glass..The glass was held vertically in place by a custom built holder. | Front two fee | Six Gecko species |
| Hagey, T.J., Uyeda, J.C., Crandell, K.E., Cheney, J.A., Autumn, K. and Harmon, L.J., 2017. Tempo and mode of performance evolution across multiple independent origins of adhesive toe pads in lizards. *Evolution*, *71*(10), pp.2344-2358. | Measure the “...angle of toe detachment. By suspending pad-bearing lizards from a glass microscope slide by a single rear toe. When the glass substrate is near vertical, the lizard's toe pad, and hence setae, are predominantly generating friction relative to the substrate. As the substrate is slowly inverted, the setae generate relatively less friction and more adhesion. At the angle of toe detachment, the setae can no longer maintain the proper orientation with the substrate to remain attached and the animal falls onto a cushioned base. | Single rear toe | three families of geckos (Gekkonidae, Phyllodactylidae, and Diplodactylidae) and the genus *Anolis* |
| Hagey, T.J., Puthoff, J.B., Holbrook, M., Harmon, L.J. and Autumn, K., 2014. Variation in setal micromechanics and performance of two gecko species. *Zoomorphology*, *133*(2), pp.111-126. | To measure toe detachment angle (TAD), we suspended a live, non-sedated gecko from a single rear, middle toe from a clean glass microscope slide using the animal’s natural clinging ability | Single rear toe | *Phelsuma grandis, Gecko gecko* |
| Higham, T.E., Gamble, T. and Russell, A.P., 2017. On the origin of frictional adhesion in geckos: small morphological changes lead to a major biomechanical transition in the genus Gonatodes. Biological Journal of the Linnean Society, 120(3), pp.503-517. | “We measured the adhesive force of each lizard by hanging a clear section of acrylic from the output hook of a force meter. Maximum tensile frictional adhesive force of the forelimbs was quantified by allowing the animal to attach and then pulling it away from, and in line with, the output hook. Animals were slowly pulled by hand and moved no more than 3 cm. | Front two feet | *Gonatodes humeralis* |
| Irschick, D. J. et. al 1996. A comparative analysis of clinging ability among pad-bearing lizards. *Biological Journal of the Linnean Society*, *59*(1), 21–35. https://doi.org/10.1006/bijl.1996.0052 | Measured the “vertical force required to pull a lizard down a standard, nearly vertical (85° to the horizontal) surface (acetate overhead transparency sheet attached to and overlying a stiff plexiglass plate)” | Front two feet | 14 species of pad-bearing lizards within the families Gekkonidae, Scincidae and Polychridae |
| Irschick, D.J., Vanhooydonck, B., Herrel, A. and Meyers, J.A.Y., 2005. Intraspecific correlations among morphology, performance and habitat use within a green anole lizard (Anolis carolinensis) population. Biological Journal of the Linnean Society, 85(2), pp.211-221. | Lizards adhere to an acetate sheet attached to the surface of the force plate with tape. During a trial, the lizard was removed from the incubator and placed with its front feet on the acetate sheet (see below). It was then repeatedly dragged horizontally at a constant speed (i.e. not jerked) across the force plate for 30 s | Front two feet | Anolis carolinensis |
| Kolbe, J. J. (2015). Effects of Hind-Limb Length and Perch Diameter on Clinging Performance in Anolis Lizards from the British Virgin Islands. Journal of Herpetology, 49(2), 284–290. https://doi.org/10.1670/13-104 | “Lizards  were positioned so that all four limbs wrapped around the perch  rather than being held close to their body. I attached the harnessed lizard to a digital force gauge and then moved the perch and  lizard at a slow, constant speed away from the force gauge such that the perch and lizard were oriented vertically and perpendicular to the sensing head of the force gauge.” | All four feet | Anolis lizards |
| Losos, J.B., Walton, B.M. and Bennett, A.F., 1993. Trade-offs between sprinting and clinging ability in Kenyan chameleons. Functional Ecology, pp.281-286. | “Clinging force was measured by suspending a horizontal bar from a 500-g Pesola scale. A chameleon was grasped dorsally and allowed to grab onto the bar with one hindfoot. The chameleon then slowly moved downward, pulling the bar down with it. The maximum clinging force was registered on the Pesola scale when the lizard could no longer hold onto the bar.” | Hind foot | *Chamaeleo dilepis*  *Chamaeleo jacksonii* |
| Naylor, E. R., & Higham, T. E. (2019). Attachment Beyond the Adhesive System: The Contribution of Claws to Gecko Clinging and Locomotion. Integrative and Comparative Biology, 59(1), 168–181. https://doi.org/10.1093/icb/icz027 | “Geckos freely placed the right  manus onto a test surface affixed to a portable force  gauge (Mark-10 Series 5) and were steadily pulled in  parallel opposition until slipping occurred. | Front right foot | Geckos |
| O’Donnell, M. K., & Deban, S. M. (2020). Cling performance and surface area of attachment in plethodontid salamanders. The Journal of Experimental Biology, 223. https://doi.org/10.1242/jeb.211706 | Salamanders were “placed on a clear, dry sheet of acrylic fastened on a rotating hinge of adhesive tape to a laboratory table edge at an angle of 0 deg relative to horizontal.” | All four feet | 20 species of Plethodontid salamanders |
| Pillai, R., Nordberg, E., Riedel, J., & Schwarzkopf, L. (2020). Nonlinear variation in clinging performance with surface roughness in geckos. Ecology and Evolution, 10(5), 2597–2607. https://doi.org/10.1002/ece3.6090 | “Attached a force gauge to the inguinal region of the gecko using a harness of fishing line.Geckos were then pulled horizontally backward at an angle of 0° relative to the tabletop, using a constant velocity (~0.5 cm/s, calibrated using a 30-cm ruler and stopwatch.” | All four feet | ‘Two Diplodactylid gecko species  (*Oedura cogger*i, and *Pseudothecadactylus australis*)’ |
| Pillai, Rishab et al. “Geckos Cling Best to, and Prefer to Use, Rough Surfaces.” Frontiers in zoology 17.1 (2020): 32–32. Web. | “Attached a force gauge to the inguinal region of the gecko using a harness of fishing line.Geckos were then pulled horizontally backward at an angle of 0° relative to the tabletop, using a constant velocity (~0.5 cm/s, calibrated using a 30-cm ruler and stopwatch.” | All four feet | Oedura  geckos |
| Schwarz, R., Stark, G., Antonopolous, A., Itescu, Y., Pafilis, P., Chapple, D.G. and Meiri, S., 2021. Specialist versus generalist at the intraspecific level: functional morphology and substrate preference of Mediodactylus kotschyi geckos. *Integrative and Comparative Biology*, *61*(1), pp.62-75. | “Attached each gecko to a 50 g Pesola spring balance by a harness placed around its abdomen. We then placed the gecko onto the test substrate and drew the scale horizontally backward at an even speed.” | All four feet | *Mediodactylus kotschyi* |
| ZANI, P., 2000. The comparative evolution of lizard claw and toe morphology and clinging performance. *J. EVOL. BIOL*, *1*(3), pp.316-325. | Attached a “A pesola spring loaded balance around the waist of the animal. The lizard was then placed on a horizontal substrate. I slowly (approximately 0.5 m s±1) drew the balance horizontally away from the posterior of the animal and recorded the maximum force required to pull the stationary animal from the substrate.” | All four feet | Sauromalus obesus, Coleonyx variegatus and Sceloporus magister, |
